# Supplementary material for: NF-κB inducing kinase is a therapeutic target for systemic lupus erythematosus
Source: Nat Commun. 2018 Jan 12;9:179. doi: 10.1038/s41467-017-02672-0 (PMC5766581; doi:10.1038/s41467-017-02672-0)
Supplement: Supplementary file 1 — Supplementary Information [file 41467_2017_2672_MOESM1_ESM.pdf]

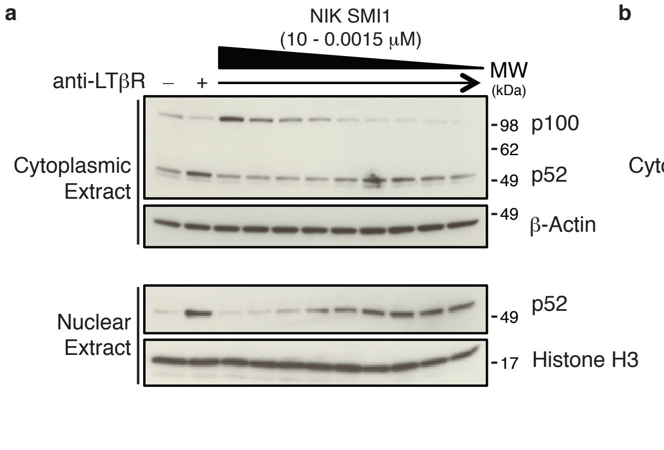

Supplementary Figure 1. NIK inhibition of non-canonical and canonical NF-κB signaling

Western blots of cytoplasmic and nuclear extracts from anti-LTβR antibody stimulated HeLa cells with serial titration of NIK SMI1 were analyzed with antibodies directed against p100/p52 (a), or RelA and phospho-RelA (b). β-Actin, Histone H3, and Lamin B1 were used as controls for cytoplasmic and nuclear extracts.

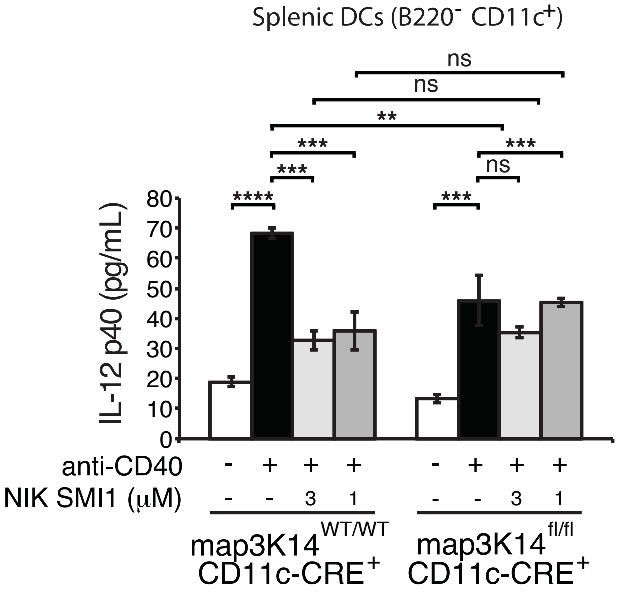

Supplementary Figure 2. NIK inhibition of IL-12p40 production from splenic DCs

Splenic DCs (B220$^-$ CD11c$^+$) were sorted from *Nik*$^{WT/WT}$, CD11c-CRE$^+$ and *Nik*$^{fl/fl}$, CD11c-CRE$^+$ mice and subsequently cultured with anti-CD40 (1 μg/mL) and NIK SMI1 (3 μM or 1 μM) for 18 hrs. IL-12p40 production was measured by ELISA. Data represented as mean ± standard deviation of 3 biological replicates. One-way ANOVA with Tukey's test for multiple comparisons. *p<0.05, **p<0.01, ***p<0.001; ****p<0.0001; ns, not significant.

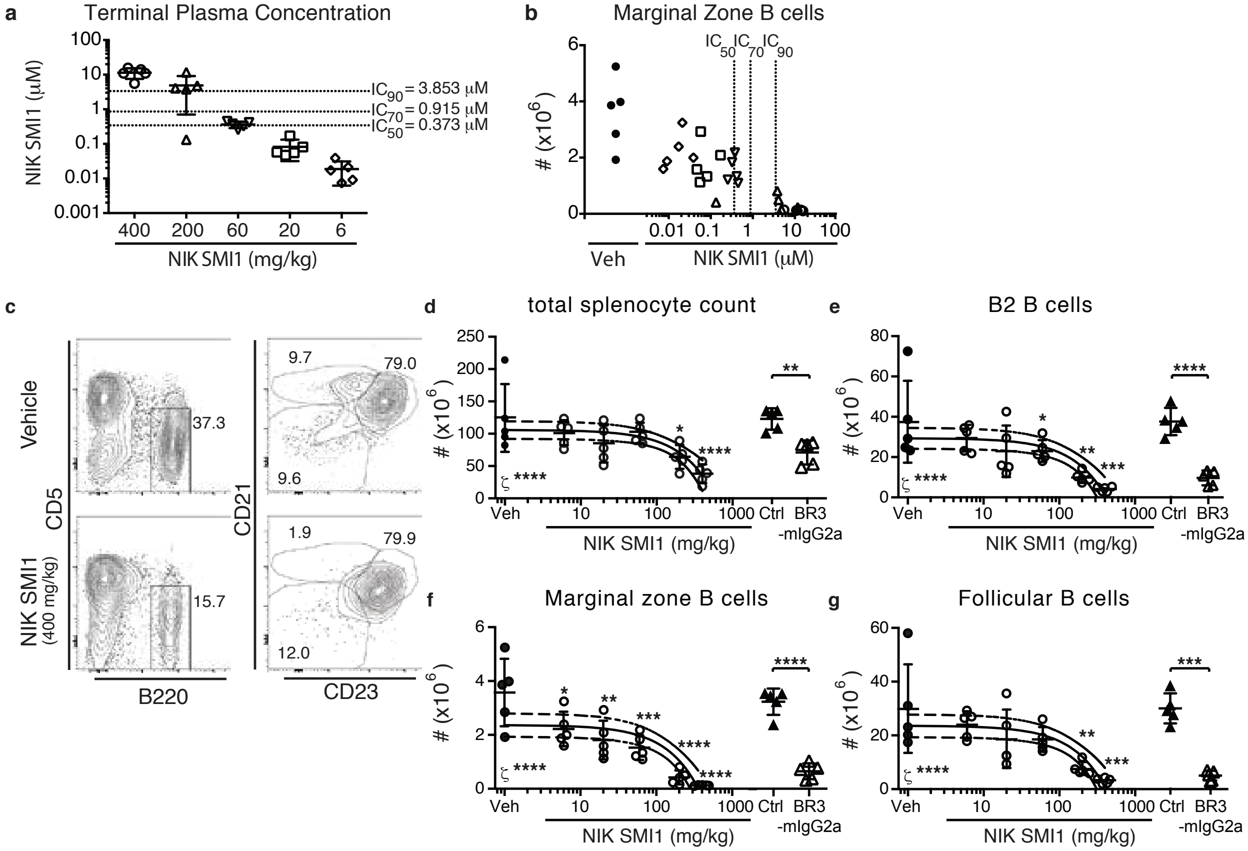

Supplementary Figure 3. PK/PD relationship of NIK SMI1

(a) Measurement of terminal plasma concentration of NIK SMI1, taken 12 hours after the final dose. (b) Correlation of terminal NIK SMI1 concentration with reduction of marginal zone B-cells. For both A and B, values for IC_{50}, IC_{70}, and IC_{90} were determined by the BAFF driven B-cell survival assay shown in Figure 2c. (c) Flow cytometry gating scheme for B cell subsets. (d-g) Dose dependent effects of NIK SMI1 on splenocyte count (d), and cellularity of B2 B cells ($B220^{+}$ $CD5^{-}$) (e), marginal zone B cells ($B220^{+}$ $CD21^{+}$ $CD23^{low/-}$) (f), and follicular B cells ($B220^{+}$ $CD21^{-}$ $CD23^{+}$) (g) within total splenocytes. (c-g) representative data from 3 independent experiments are shown. Data represented as mean ± standard deviation of 4-5 mice per group and linear regression curve (solid line) and 95% confidence interval curves (dashed lines) are drawn demonstrating statistically significant (ζ) negative slope and reduction with increasing NIK SMI1 dose. Statistics: Vehicle vs. NIK SMI1 doses, One-way ANOVA with Dunnett's test for multiple comparisons. Isotype control vs. BR3-mIgG2a, unpaired t-test with Welch's correction. * $p<0.05$, ** $p<0.01$, *** $p<0.001$, **** $p<0.0001$, ns = not significant.

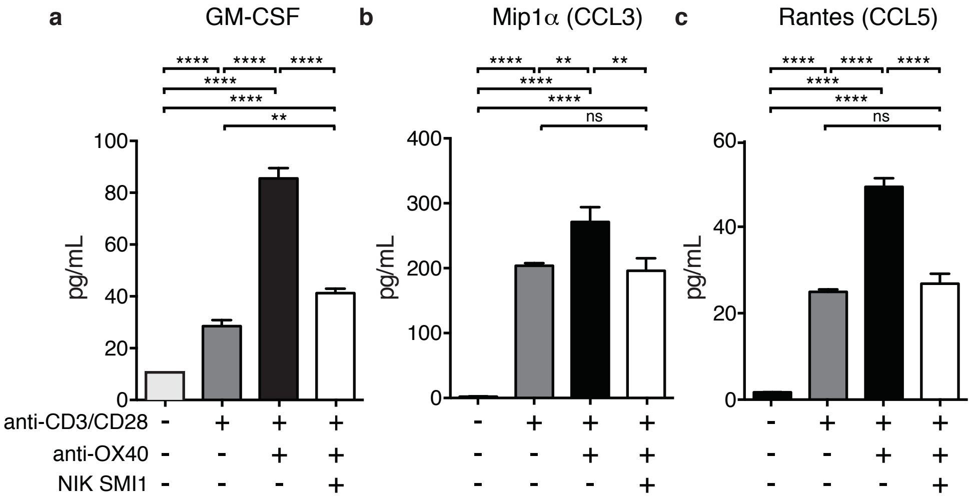

Supplementary Figure 4. Cytokine and Chemokine expression by OX40 stimulated T cells

(a-c) OX40 induced cytokine production from memory $CD4^+$ T-cells in the presence or absence of 3 μM NIK SMI1. GM-CSF (a), MIP1α (CCL3) (b), Rantes (CCL5) (c). Data are represented as mean ± standard deviation of 3 biological replicates. One-way ANOVA with Sidak's test for multiple comparisons. * $p<0.05$, ** $p<0.01$, *** $p<0.001$, **** $p<0.0001$, ns = not significant.

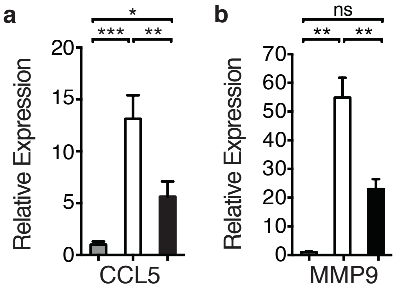

Supplementary Figure 5. Regulation of TWEAK inducible genes in human RPTEC

(a-b) qRT-PCR measurement of human CCL5 (a) and MMP9 (b) relative to HPRT. qRT-PCR data are represented as mean ± standard deviation of 3 biological replicates run in experimental duplicate. Unstimulated + DMSO (gray bar), TWEAK (100 ng/mL) + DMSO (white bar), and TWEAK (100 ng/mL) + 3 µM NIK SMI1 (black bar). Statistics: One-way ANOVA with Sidak's test for multiple comparisons. * $p<0.05$, ** $p<0.01$, *** $p<0.001$, **** $p<0.0001$, ns = not significant.

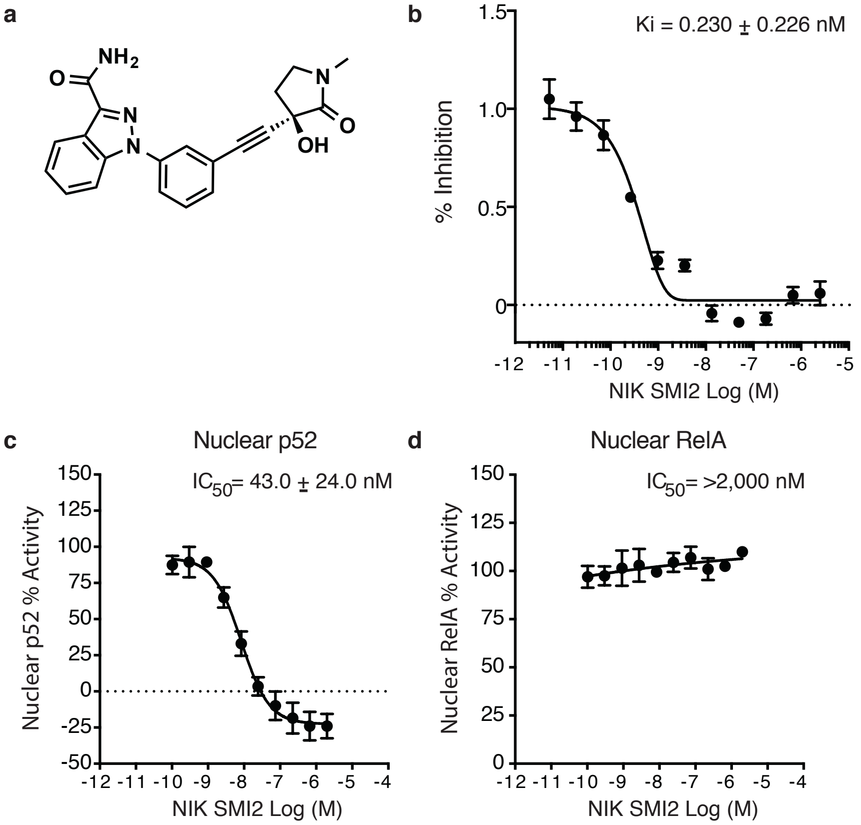

Supplementary Figure 6. Characterization of NIK SMI2

(a) Chemical structure of NIK SMI2. (b) Inhibition of human NIK enzymatic activity by SMI2. A representative curve is shown, and the calculated Ki represents the mean ± standard deviation of 2 independent experiments. (c-d) Inhibition of anti-LTβR induced non-canonical (c) and TNF induced canonical (d) NF-κB signaling by NIK SMI2. Representative curves are shown, and IC_{50} measurements represent mean ± standard deviation of 5 and 10 independent experiments, respectively.

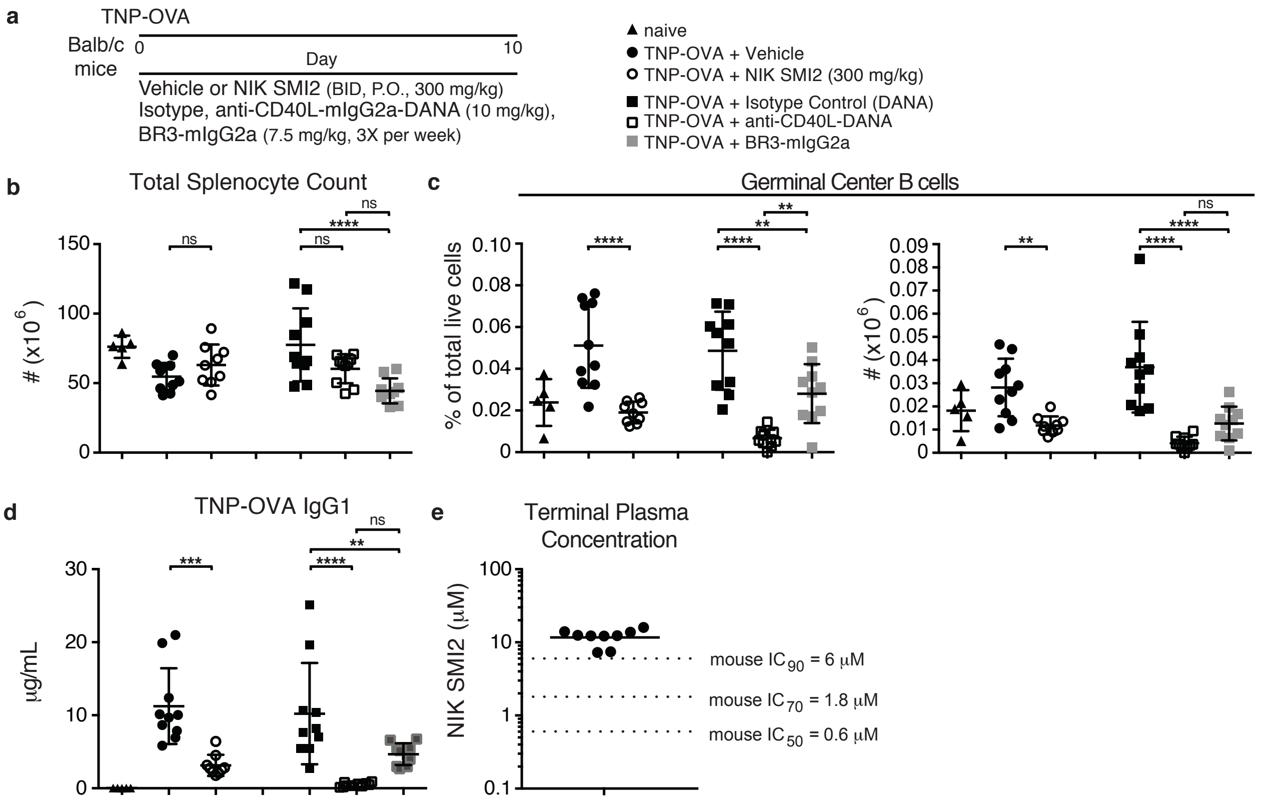

Supplementary Figure 7. NIK inhibition suppresses the humoral response

(a) Experimental Design. Balb/c mice were immunized with TNP-OVA on day 0 and treated with vehicle, NIK SMI2, anti-CD40L-mIgG2a-DANA, BR3-mIgG2a, or appropriate controls for 10 days, as indicated in the legend. (b-d) *In vivo* effect of NIK inhibition, CD40 blockade, and BAFF blockade on splenocyte count (b), Germinal center B cells ($B220^{+}$ IgM^{-} IgD^{-} $GL7^{+}$ $CD95^{+}$) percentage and number (c), and serum TNP-OVA-specific IgG1 levels (d). (e) Measurement of terminal plasma concentration of NIK SMI2, taken 12 hours after the last dose was administered. Data represent mean ± standard deviation of 5-10 Balb/c mice per group. Statistics: (b-d) One-way ANOVA with Sidak's test for multiple comparisons. * $p<0.05$, ** $p<0.01$, *** $p<0.001$, **** $p<0.0001$, ns = not significant.

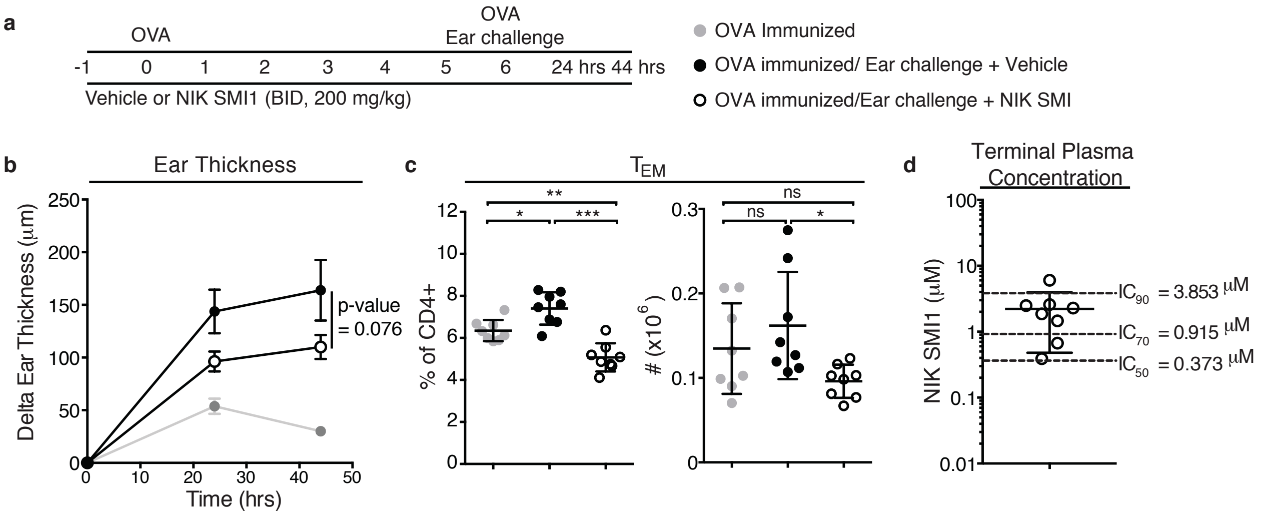

Supplementary Figure 8. Effect of NIK inhibition on the delayed type hypersensitivity response

(a) Experimental Design. C57Bl/6 mice were immunized with OVA on day 0 and challenged on day 6. Vehicle or NIK SMI1 treatment was applied throughout the experiment. (b-c) *In vivo* effect of NIK inhibition on ear thickness (b) and T effector/memory cell (T_{EM}, $CD4^{+}$ $CD44^{+}$ $CD62L^{-}$) percentage and absolute numbers (c). (d) Measurement of terminal plasma concentration of NIK SMI1, taken 12 hours after the last dose was administered. Ear thickness data represent mean ± standard error of the mean of 8 C57BL/6 mice per group. TEM and terminal plasma concentration data represent mean + standard deviation of 8 mice per group. Statistics: (b-c) One-way ANOVA with Tukey's test for multiple comparisons. * p<0.05, ** p<0.01, *** p<0.001, **** p<0.0001, ns = not significant.

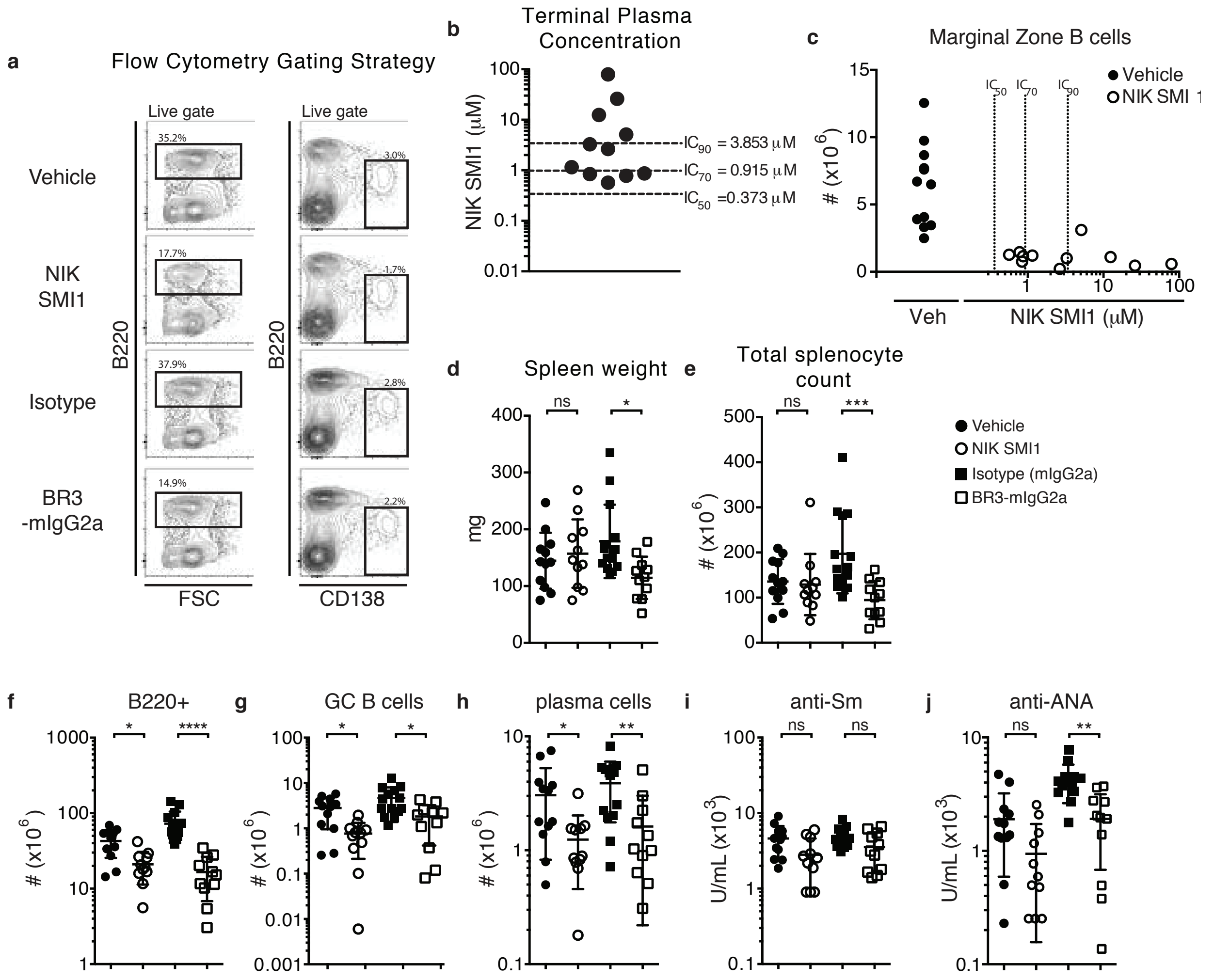

Supplementary Figure 9. NIK inhibition reduces B cell function in NZB/W F1mice

(a) Gating strategy for FACS analysis. (b) Measurement of terminal plasma concentration of NIK SMI1, taken 12 hours after the final dose was administered. (c) Correlation of NIK SMI1 exposure with reduction of marginal zone B-cells. For both (b) and (c), values for IC_{50}, IC_{70}, and IC_{90} were determined by the BAFF driven B-cell survival assay shown in Figure 2c. (d-e) Spleen weights and total splenocyte count. (f-h) Absolute cell counts corresponding to percentages depicted in Figure 5b-5d, B220$^+$ (f), germinal center B cells (g), plasma cells (h). (i-j) ELISA measurement of serum autoantibodies against Smith antigen (Sm) and anti-nuclear antigen (ANA). (d-j) data represent mean ± standard deviation of 9-11 C57BL/6 mice per group. Statistics: (d-j) Krushal-Wallis test with Dunn’s test for multiple comparisons. * $p<0.05$, ** $p<0.01$, *** $p<0.001$, **** $p<0.0001$, ns = not significant.

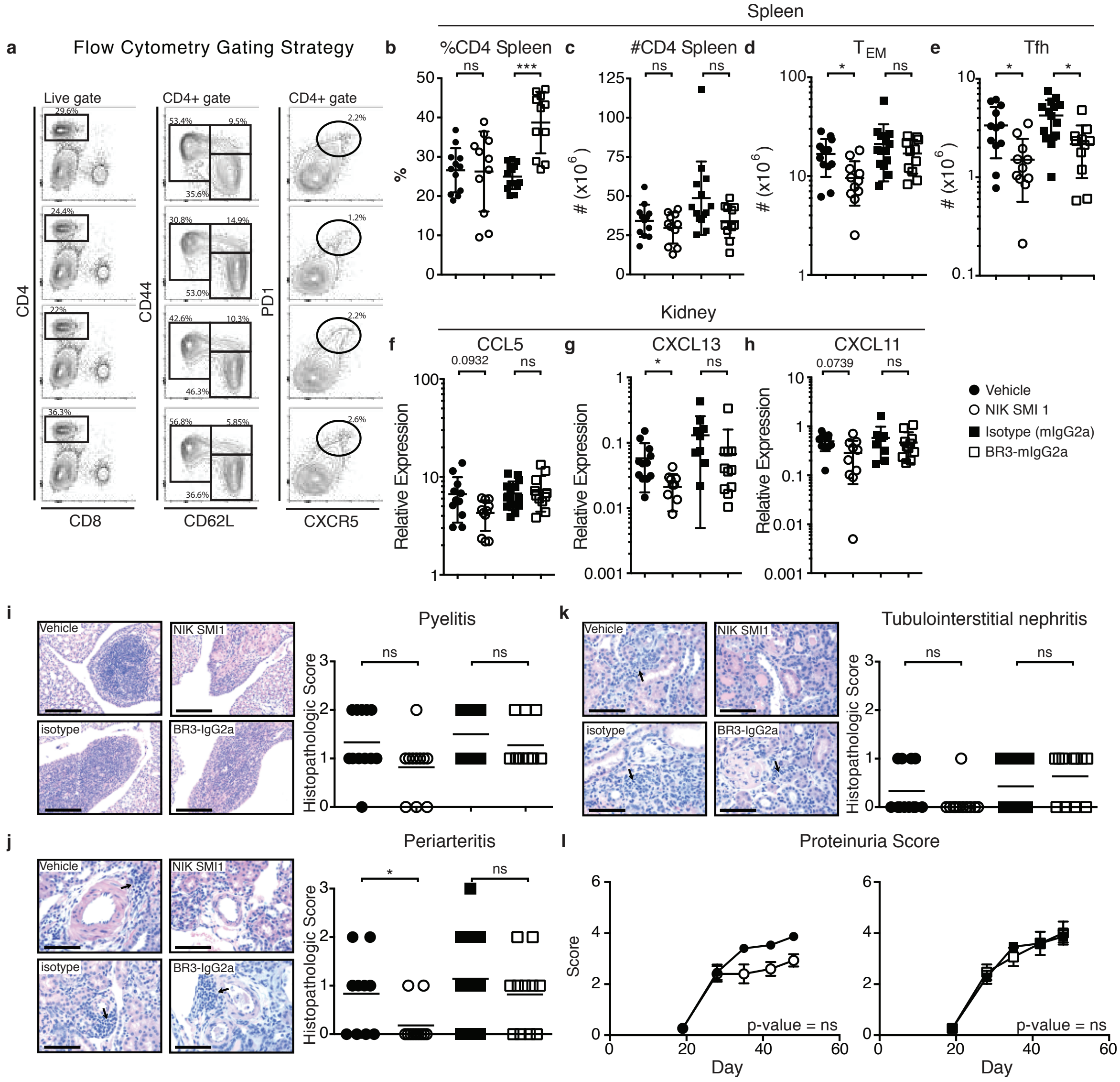

Supplementary Figure 10. NIK inhibition suppresses BAFF independent biology in NZB/W F1 mice

(a) Gating strategy for FACS analysis. (b-c) Percentage (b) and absolute number (c) of $CD4^+$ splenocytes. (d-e) Absolute numbers of T effector memory cells (d), and of T follicular helper cells (e) corresponding to percentages depicted in Figure 6 (a-b). (f-h) Gene expression of CCL5 (f), CXCL13 (g), and CXCL11 (h) in the kidney as measured by Fluidigm analysis. (i-k) Histological assessment and scoring of Pyelitis (Scale bars, 200 µm) (i), Periarteritis (j) (Scale bars, 100 µm), and Tubulo-interstitial nephritis (k) (Scale bars, 100 µm). (l) Proteinuria measurements over the course of 4 weeks. (b-h) Data represented as mean ± standard error of the mean of 9-11 C57BL/6 mice per group. Statistics: (b-h) Krushal-Wallis test with Dunn's test for multiple comparisons. (i-j) histopathology, non-adjusted p-values were generated using Fisher's exact test; (l) for proteinuria, area under the curve (AUC) from day 19-49 was calculated for each animal, and groups were compared by one-way ANOVA with Sidak's test for multiple comparisons. * p<0.05, ** p<0.01, *** p<0.001, ns = not significant.

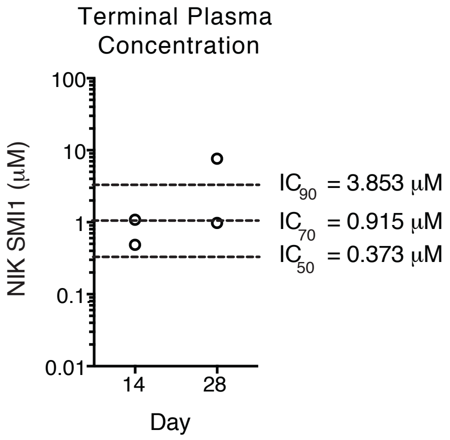

Supplementary Figure 11. Exposure of NIK SMI1 during the IFN✔ -accelerated NZB/ W F1 efficacy study

Representative measurement of NIK SMI1 plasma concentration from day 14 and day 28 taken at the end of the dosing interval of 12 hours. Limited analysis was done so as not to interfere with efficacy determination in experimental groups. IC_{50}, IC_{70}, and IC_{90} values as determined by BAFF induced mouse B cell survival assay (Figure 2c).

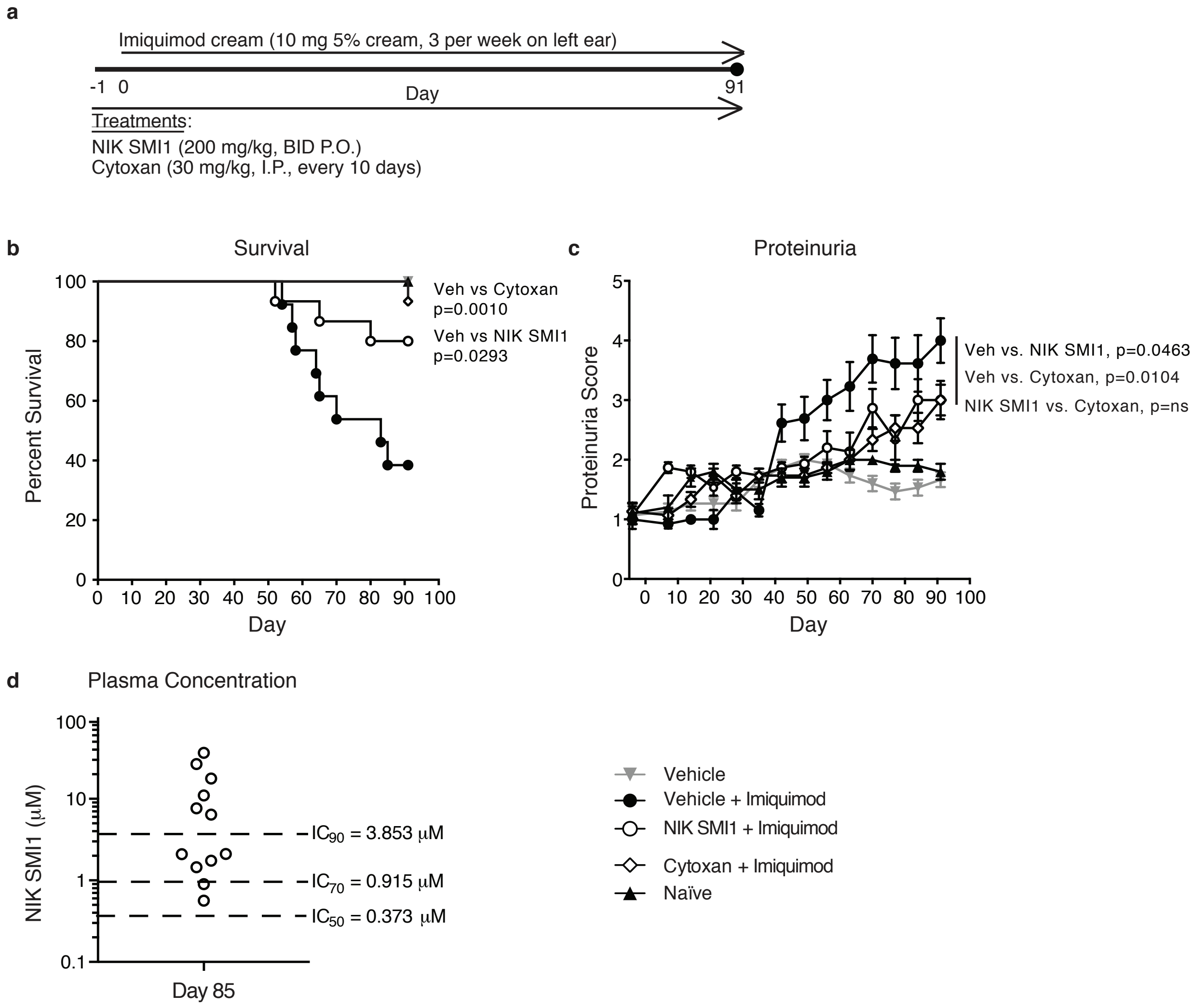

Supplementary Figure 12. Effect of NIK inhibition and Cytoxan on survival and proteinuria in Imiquimod treated FVB mice

(a) Experimental design. FVB mice were treated with Imiquimod and NIK SMI1, Cytoxan or vehicle control for 91 days. (b-c) Kaplan-Meier curve of percent survival (b) and proteinuria score (c) of NIK inhibitor or Cytoxan treated Imiquimod-FVB mice and controls. Proteinuria data are represented as mean ± standard error of the mean of 15 mice for each time point. For deceased mice, a proteinuria score of 5 was carried forward. Statistics: Survival, Mantel-Cox Test; For proteinuria, area under the curve (AUC) from day 42-91 was calculated for each animal, and groups were compared by one-way ANOVA with Sidak's test for multiple comparisons. (d) Measurement of plasma concentration of NIK SMI1 on day 85, taken 12 hours after the final dose.

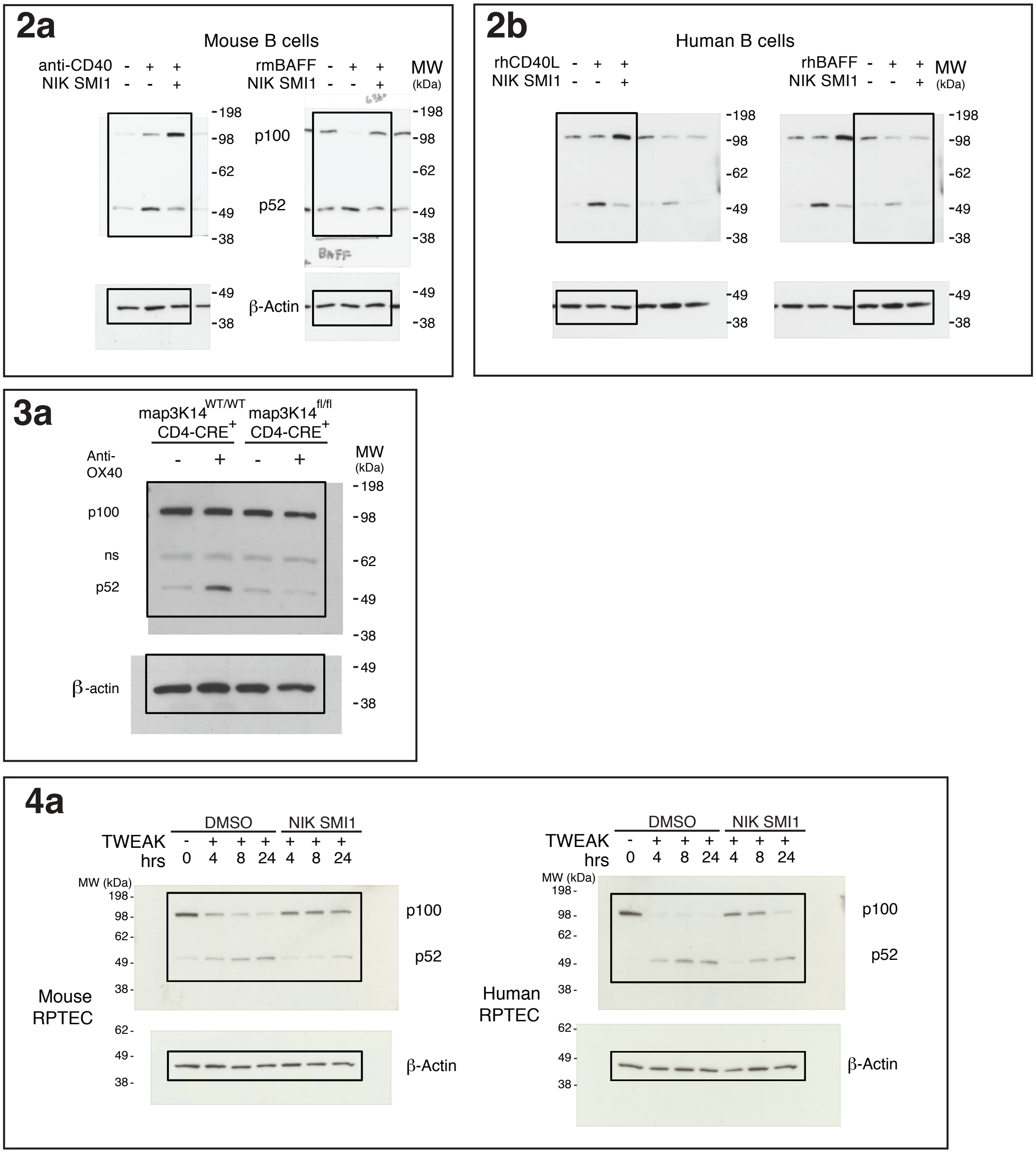

Supplementary Figure 13. Uncropped images for Figures 2-4.

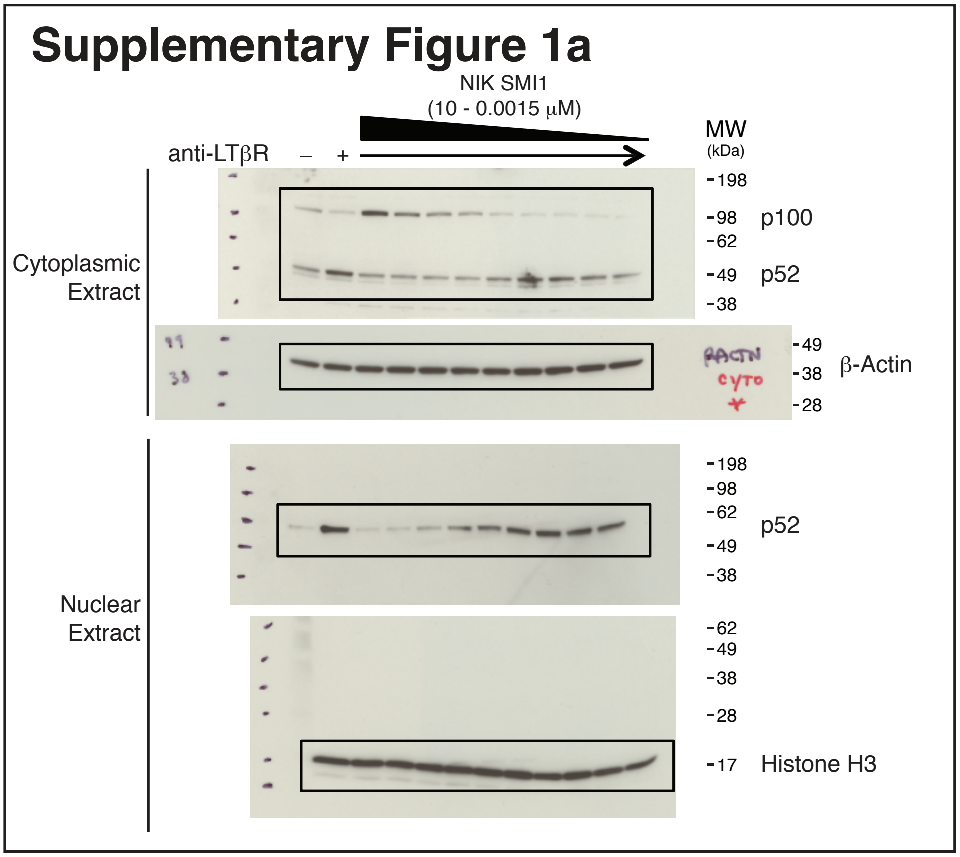

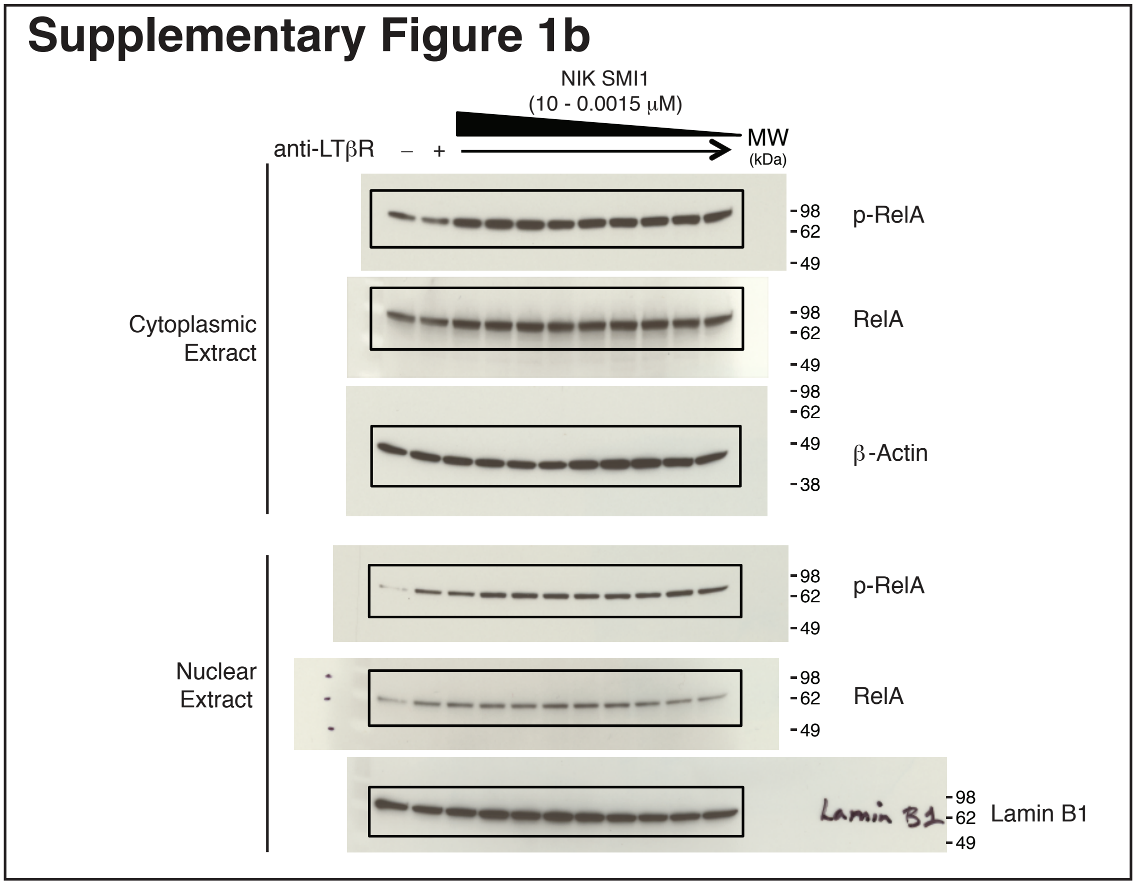

Supplementary Figure 14. Uncropped images for Supplementary Figure 1

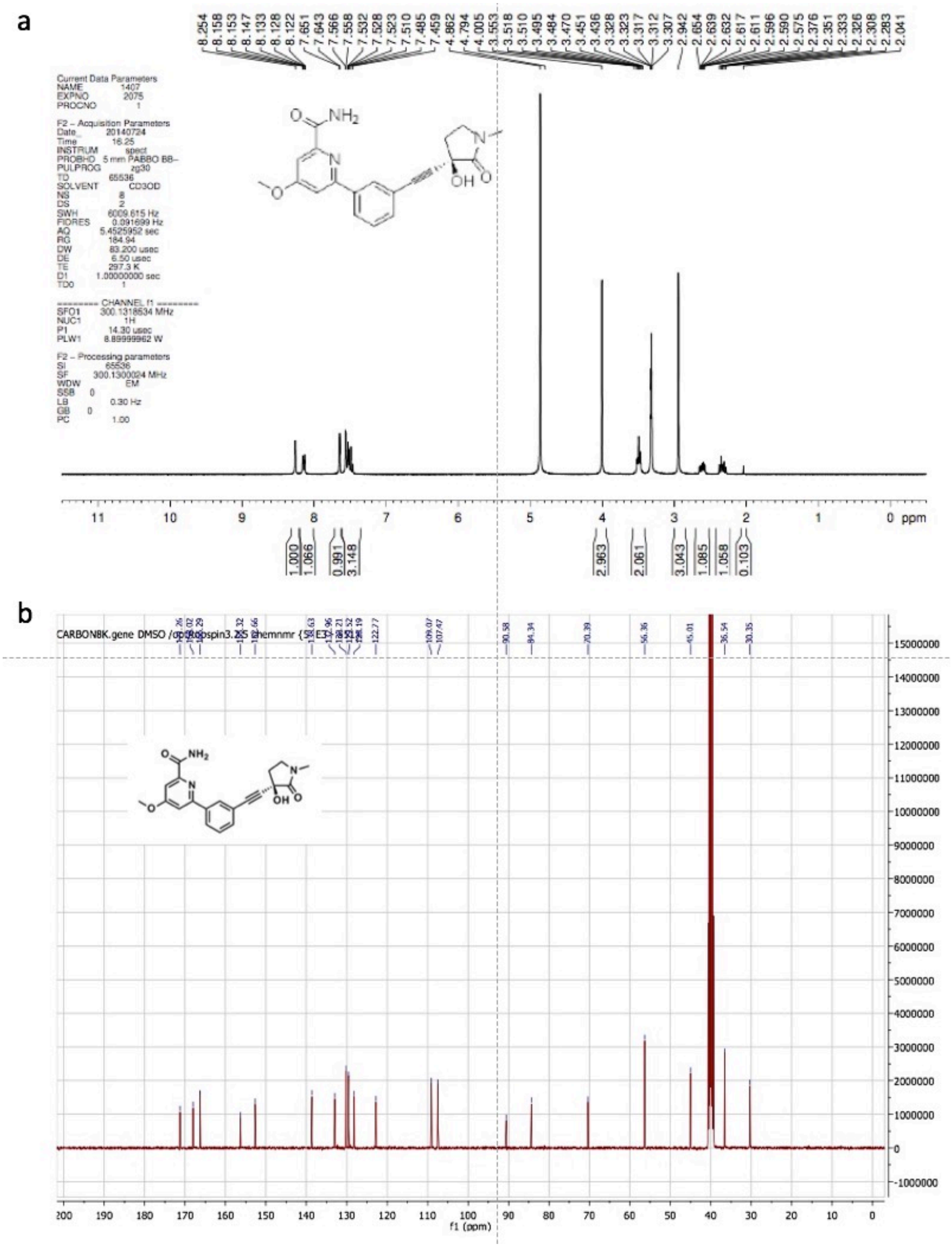

Supplementary Figure 15. ^{1}H NMR spectra (a) and ^{13}C NMR spectra (b) of NIK SMI1

a

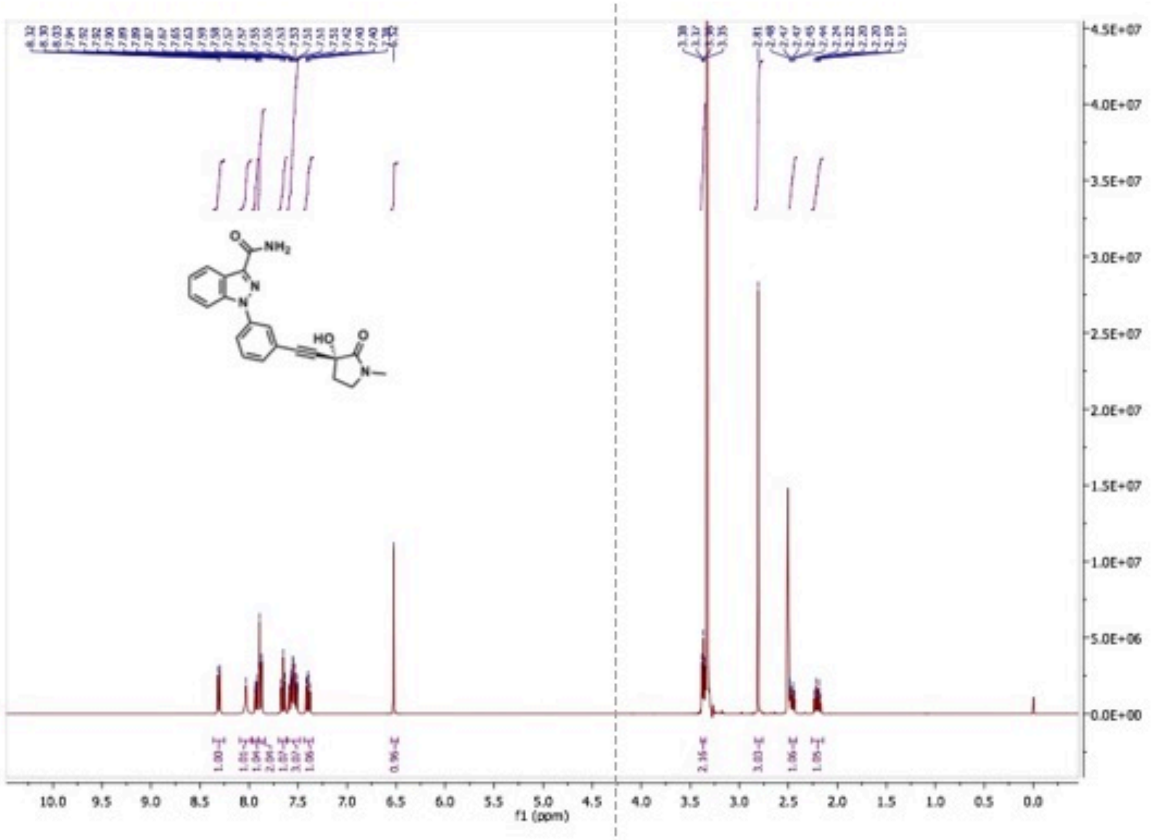

b

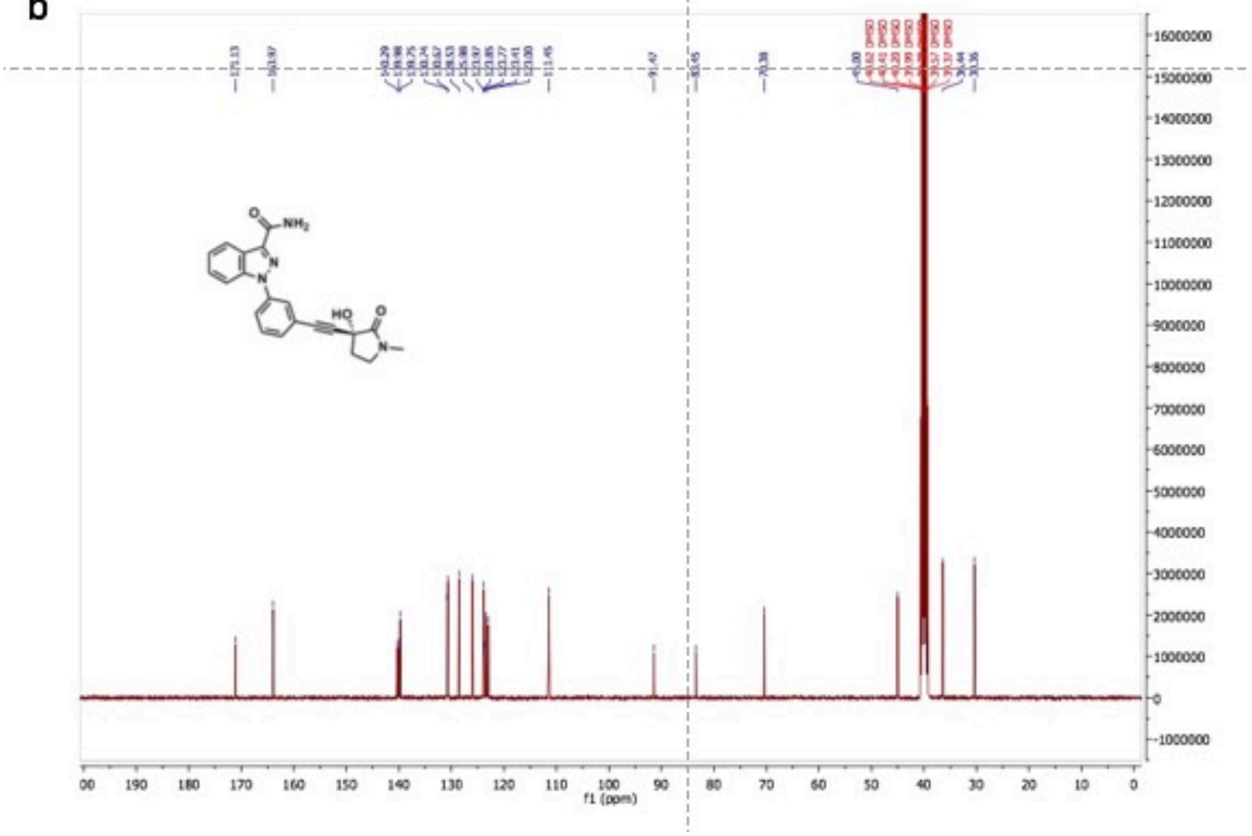

Supplementary Figure 16. ^{1}H NMR spectra (a) and ^{13}C NMR spectra (b) of NIK SMI2

Kinase	[ATP]	NIK SMI1 %	Kinase	[ATP]	NIK SMI1 %	Kinase	[ATP]	NIK SMI1 %	Kinase	[ATP]	NIK SMI1 %
NIK*	NA	98.8	AKT2	Km app	13	SIK2	Km app	5.5	STK16	NA	1
KHS1	Km app	96	Ron	Km app	13	EphA7	Km app	5.1	AVCR2B	NA	0.8
LRRK2	Km app	82.1	CSK	Km app	12.5	GSK3_beta	Km app	5	GRK5	Km app	0.5
PKD1	Km app	79	Ros	Km app	12.5	IRR	Km app	5	Lck	Km app	0.5
MEKK2	NA	66.8	P38_delta	Km app	12.5	RSK3	Km app	5	MRCK_alpha	Km app	0.5
PAK4	Km app	64	Rse	Km app	12	SGK1	Km app	5	Yes	Km app	0.5
TSSK1	Km app	61	Srm	Km app	12	JNK3	NA	4.6	TNK2	NA	0.1
Mink1	Km app	53.5	MSSK1	Km app	11.5	CHK2	Km app	4.5	Met	Km app	0
MAP4K4	Km app	45.5	MST1	Km app	11.5	CK2_alpha1	Km app	4.5	PKD2	Km app	0
MYLK3	NA	45.3	MEK3	NA	11.4	CLK1	Km app	4.5	RIPK2	NA	-0.1
MEK1	NA	43.1	NEK4	Km app	11	DYRK1A	Km app	4.5	B-Raf	NA	-0.3
YSK1	Km app	39	PrKX	Km app	11	GRK2	Km app	4.5	PDK1	Km app	-0.5
PKC_eta	Km app	38	FAK	Km app	10.5	HIPK1	Km app	4.5	PIM1	Km app	-0.5
Cot	100	34.5	MLK2	NA	9.9	Hyl	Km app	4.5	SGK3	Km app	-0.5
MST4	Km app	33.5	Abl	Km app	9.5	IKK_beta	Km app	4.5	Syk	Km app	-0.5
MST3	Km app	32.5	EGFR (T790M,L858R)	Km app	9.5	PKC_alpha	Km app	4.5	ZAK	NA	-0.8
NEK1	Km app	31.5	ROCK1	Km app	9.5	RSK1	Km app	4.5	LTK	Km app	-1
PAK6	Km app	31.5	SGK2	Km app	9.5	TXK	Km app	4.5	MLK1	Km app	-1
TAK1-TAB1	NA	31.1	p70S6K	Km app	9.5	NLK	NA	4.2	mTOR	Km app	-1
CaMKII_beta	Km app	30.5	Axl	Km app	9.3	PLK2	Km app	4	CDK2/CyclinA	Km app	-1.5
AKT1	Km app	28.5	Flt4	Km app	9	eEF-2K	Km app	4	ErbB2	Km app	-1.5
MST2	Km app	28	GRK6	Km app	9	STK33	NA	3.9	IRAK4	Km app	-1.5
MELK	Km app	26.5	PAK1	Km app	9	ASK1	NA	3.8	KDR	Km app	-1.5
TBK1	Km app	26.5	PLK3	Km app	9	Mer	Km app	3.5	RAF1 (Y340D, Y341D)	NA	-1.5
CLK2	Km app	25	P38_beta	Km app	9	DRAK1	NA	3.4	Src	Km app	-1.5
Aurora_B	Km app	24.5	CDK1/cyclinB	Km app	8.5	IKK_alpha	Km app	3.1	PI3K-G	Km app	-2.9
MKNK2	NA	23.3	CK1_gamma2	Km app	8.5	HIPK4	Km app	3	BTK	Km app	-3
SPHK1	Km app	21.3	IGF1R	Km app	8.5	JNK2	NA	3	ITK	Km app	-3
Fes	Km app	20.5	JAK2	Km app	8.5	DDR1	NA	2.7	PKA	Km app	-3
PRKAA1	Km app	20.5	SRPK1	Km app	8.5	SLK	NA	2.6	PhK_gamma2	Km app	-3
IKK_epsilon	Km app	19	Fgr	Km app	8	CDK5/p25	Km app	2.5	EphA8	Km app	-3.5
CaMKI	100	18.3	CDK8/cyclinC	NA	7.8	CK1_gamma1	Km app	2.5	FGFR3	Km app	-3.5
Frk	Km app	18	FGFR4	Km app	7.5	EphA1	Km app	2.5	Kit	Km app	-3.5
CamKII_alpha	Km app	17.5	MARK3	Km app	7.5	Lyn	Km app	2.5	PAK3	Km app	-3.5
HIPK2	Km app	17.5	MSK1	Km app	7.5	TrkA	Km app	2.5	TrkB	Km app	-3.5
NEK6	Km app	17	MYLK	NA	7.5	ACVR1B	Km app	2	BMPR1A	NA	-3.9
TAO1	Km app	17	ROCK2	Km app	7.5	CK1_alpha1	Km app	2	DNA-PK	Km app	-4
ZAP-70	Km app	17	Brk	Km app	7	InsR	Km app	2	CK1_delta	Km app	-4.5
LIMK1	NA	16.8	EphB1	Km app	7	MKNK1	Km app	2	PDGFR_alpha	Km app	-4.5
MARK1	Km app	16	ErbB4	Km app	7	PKC-epsilon	Km app	2	Bmx	Km app	-5
PASK	Km app	16	Flt1	Km app	7	Ret	Km app	2	MAPKAPK2	Km app	-5
CDK9/cyclinT1	Km app	15.3	PKC_zeta	Km app	7	TYK2	Km app	2	EphA3	NA	-5.2
DAPK1	Km app	15.1	RSK2	Km app	7	IRAK1	Km app	1.8	CDK7/cyclinH	Km app	-5.5
CaMKI_delta	Km app	15	CK1_epsilon1	Km app	6.5	WNK2	NA	1.7	FGFR1	Km app	-5.5
GSK3_alpha	Km app	15	DCAMKL2	Km app	6.5	DMPK	NA	1.6	ZIPK	Km app	-5.5
JAK3	Km app	15	PhK_gamm1	Km app	6.5	ARK5	Km app	1.5	PKC_theta	Km app	-6
CLK3	Km app	14.5	WEE1	NA	6.2	DYRK3	Km app	1.5	JAK1	Km app	-7
CamKIV	Km app	14.5	MuSK	Km app	6	GRK3	Km app	1.5	PI3K-A	Km app	-7.5
PKG1_alpha	Km app	14.5	PRAK	Km app	6	NEK9	Km app	1.5	p38_alpha	Km app	-7.5
CHK1	Km app	14	Tie2	Km app	6	PKC_beta1	Km app	1.5	TGFBR1	NA	-9.4
P38_gamma	Km app	14	ALK2	NA	5.8	PKD_delta	Km app	1.5	Blk	Km app	-10.5
CLK4	NA	13.7	JNK1_alpha1	100	5.6	PLK1	Km app	1.5	CSF1R	Km app	-11
BrSK1	Km app	13.5	DYRK4	Km app	5.5	CAMKK2	NA	1.1	EGFR	Km app	-11.5
Flt3	Km app	13.5	ERK2	Km app	5.5	TEC	NA	1.1	CAMKK1	NA	-11.6
MKK6	NA	13.2	EphB3	Km app	5.5	Aurora_A	Km app	1			
TTK	NA	13.2	PRK1	Km app	5.5	MAPKAPK3	Km app	1			

Supplementary Table 1. Kinase selectivity of NIK SMI1 against 222 kinase panel
NIK SMI1 was used at 1 µM, except in the NIK kinase assay, where the concentration was 0.1 µM. Red, > 90% inhibition; orange, 70 - 90% inhibition; yellow, 50 - 70% inhibition; green, <50% inhibition by NIK SMI1. Km app, apparent Michaelis constant; NA, competitive binding assay containing no ATP, using a tracer instead.

**Assay**		**NIK SMI1 Potency**	**NIK SMI2 Potency**
Human NIK Enzymatic assay	K_i, nM	0.230 ± 0.170 n=2	0.230 ± 0.226 n=2
Mouse NIK Enzymatic assay	K_i, nM	0.395 ± 0.226 n=4	0.84 n=1
HeLa anti-LTβR nuclear p52	IC_{50}, nM	70.0 ± 48.0 n=5	43.0 ± 24.0 n=5
HeLa anti-LTβR nuclear RelA	IC_{50}, nM	>2000 n=8	>2000 n=10
Human B cell survival	IC_{50}, nM	189 ± 80.4 n=3	360 ± 122 n=2
Mouse B cell survival	IC_{50}, nM	373 ± 64 n=2	1748 ± 573 n=4
Human B cell anti-CD40 induced ICOSL	IC_{50}, nM	84.6 ± 50.0 n=4	58.7 ± 50.6 n=3
Human Monocyte Derived DC anti-CD40 induced IL-12p40	IC_{50}, nM	95.8 ± 9.0 n=3	60.7 ± 39.3 n=5
Human B cell anti-IgM proliferation	IC_{50}, nM	>10,000 n=3	>10,000 n=2
Human B cell anti-CD40 proliferation	IC_{50}, nM	>10,000 n=2	>10,000 n=2

Supplementary Table 2. Molecular and cellular potency of NIK SMI1

Data are represented as mean + standard deviation of the average for the number (n) of experiments conducted. For assay conditions and details, please refer to methods and materials.

Target	Species	Clone	Fluorochrome	Vendor	Method
B220	Mouse	RA3-6B2	Pacific Blue	BD Biosciences	FACS
b-Actin	Mouse/Human	polyclononal	N/A	Cell Signaling Technology	Western
BR3-mIgG2a	Mouse	N/A	N/A	Genentech, Inc	in vivo
CD4	Mouse	RM4-5	eFluoro450	eBiosciences	FACS
CD5	Mouse	53-7.3	PerCP-Cy5.5	eBiosciences	FACS
CD8	Mouse	53-6.7	FITC	BD Biosciences	FACS
CD20	Human	2H7	APC	BD Biosciences	FACS
CD21	Mouse	7G6	FITC	BD Biosciences	FACS
CD23	Mouse	B3B4	PE	BD Biosciences	FACS
CD24	Mouse	M1/69	PerCP-Cy5.5	BD Biosciences	FACS
CD38	Mouse	90	AF700	eBiosciences	FACS
CD40	mouse	37.51	N/A	BD Biosciences	in vitro stimulation
CD40L	mouse	N/A	N/A	Genentech, Inc	in vivo
CD44	Mouse	1M7	FITC	BD Biosciences	FACS
CD44	Mouse	1M7	APC	BD Biosciences	FACS
CD62L	Mouse	MEL-14	PE	BD Biosciences	FACS
CD95	Mouse	Jo2	PE-Cy7	BD Biosciences	FACS
CD138	Mouse	281-2	PE	BD Biosciences	FACS
CXCR5	Mouse	SPRCL5	PE	eBiosciences	FACS
GL7	Mouse	GL7	Biotin	eBiosciences	FACS
Histone H3	Mouse/Human	polyclononal	N/A	Cell Signaling Technology	Western
ICOS	Mouse	C398.4A	PE-Cy7	BD Biosciences	FACS
ICOSL	Human	MIH12	Biotin	eBiosciences	FACS
IgD	Mouse	11-26c.2a	APC-Cy7	BD Biosciences	FACS
IgM	Mouse	11/41	FITC	BD Biosciences	FACS
IgM	Mouse	11/41	APC	Biolegend	FACS
IgM	Human	polyclononal	N/A	Southern Biotech	in vitro stimulation
Lamin B1	Mouse/Human	polyclononal	N/A	Cell Signaling Technology	Western
LTbR	Human	polyclononal	N/A	R&D Systems	HeLa p52 High Content imaging
p52/p100	Human	05-361	N/A	Millipore	HeLa p52 High Content imaging
p52/p100	Mouse/Human	polyclononal	N/A	Cell Signaling Technology	Western
PD1	Mouse	J43	APC	eBiosciences	FACS
RelA	Mouse/Human	polyclononal	N/A	Santa Cruz	HeLa p52 High Content imaging
RelA-phospho	Mouse/Human	polyclononal	N/A	Cell Signaling Technology	Western
Streptavidin	N/A	N/A	V500	BD Biosciences	FACS
Streptavidin	N/A	N/A	PE	BD Biosciences	FACS

Supplementary Table 3 Commercial antibodies used in these studies

Gene	Species	Probe Set	Vendor
AID (ACDIA)	Human	Mm01184115_m1	Life Technologies
CCL2	Mouse	Mm00441242_m1	Life Technologies
CCL3	Mouse	Mm00441259_g1	Life Technologies
CCL5	Mouse	Mm01302427_m1	Life Technologies
CCL5	Human	Hs00982282_m1	Life Technologies
CCL9	Mouse	Mm00441260_m1	Life Technologies
CXCL11	Mouse	Mm00444662_m1	Life Technologies
CXCL13	Mouse	Mm04214185_s1	Life Technologies
CXCL16	Mouse	Mm00469712_m1	Life Technologies
CXCR5	Mouse	Mm00432086_m1	Life Technologies
GAPDH	Mouse	Mm99999915_g1	Life Technologies
HPRT	Mouse	Mm03024075_m1	Life Technologies
HPRT	Human	Hs02800695_m1	Life Technologies
ICAM1	Mouse	Mm00516023_m1	Life Technologies
ICOS	Mouse	Mm00497600_m1	Life Technologies
ICOSL	Mouse	Mm00497237_m1	Life Technologies
IgJ (J chain)	Mouse	Mm00461780_m1	Life Technologies
IL-21	Mouse	Mm0051764_m1	Life Technologies
MMP9	Mouse	Mm00442991_m1	Life Technologies
MMP9	Human	Hs00234579_m1	Life Technologies
RPL19	Mouse	Mm02601635_g1	Life Technologies
TNF	Mouse	Mm00443258_m1	Life Technologies
TWEAKR (Fn14)	Human	Mm01302476_s1	Life Technologies
VCAM1	Mouse	Mm01320970_m1	Life Technologies

Supplementary Table 4. Taqman gene expression assay primer-probe sets used for these studies

Supplementary Methods

Synthesis of NIK SMI1

Step 1: Potassium (S)-trifluoro(3-((3-hydroxy-1-methyl-2-oxopyrrolidin-3yl)ethynyl)phenyl)borate.

The following reaction was run in two equal batches: A solution of KHF_2 (66.8 g, 855 mmol, 4.0 equivalents) in water (212 mL) over 5 min to a solution of 3-iodophenylboronic acid (53 g, 213 mmol) in MeOH (424 mL). The gel/suspension was stirred at room temperature (RT) for 2h. The combined reactions were concentrated to remove methanol and placed in the fridge (2°C) for 18 h. The solid was collected by filtration and washed with cold water (2x 300 mL), hexane (3 x 100 mL) and the solid dried under vacuum for 2 days to give a total of 141 g of potassium trifluoro-(3-iodophenyl)boranide as a white solid; ^{1}H nuclear magnetic resonance (NMR) (DMSO-d_6) δ 7.62 (s, 2H), 7.39 (d, *J* = 7.1 Hz, 1H), 7. 30 (d, *J* = 7.1 Hz, 1H), 6.92 (t, *J* = 7.5 Hz, 1H).

The following reaction was run in two equal batches: A suspension of potassium trifluoro-(3-iodophenyl)boranide (29.75 g, 96 mmol), (*R*)-3-ethynyl-3-hydroxy-1-methylpyrrolidin-2-one (14.7 g, 106 mmol, 1.1 equivalents) and CuI (2.01 g, 10.6 mmol, 0.11 equivalents) in *N*,*N*-dimethylformamide (DMF) (147 mL) was treated with $Pd(PPh_3)_2Cl_2$ (7.4 g, 10.6 mmol, 0.11 equivalents). The reaction mixture was sparged with nitrogen for 5 min. Triethylamine was then added (74 mL, 528 mmol, 5.5 equivalents). The light brown solution was heated at 40°C for 1.5 h, and subsequently concentrated and azeotroped with heptanes (2x 250 mL). Water was added (350 mL) and stirred for 15 min, then the precipitate was filtered, washed with water (2 x 50 mL) and the filtrate was concentrated. The mixture was azeotroped with acetone (2x 250 mL) and dichloromethane (2 x 250 mL). Trituration with dichloromethane (DCM) (500 mL) for 1h delivered a solid that was filtered, washed with dichloromethane (2 x 100 mL) and dried under vacuum for 18 h. The combined reactions gave 55.1 g (90%) of potassium (*S*)-trifluoro(3-((3-hydroxy-1-methyl-2-oxopyrrolidin-3yl)ethynyl)phenyl)borate as a pale orange solid. Liquid chromatography–mass spectrometry (LC-MS) (Electrospray (ES), m/z): 280 $[M-K]^-$; ^{1}H NMR (DMSO-d_6) δ 7.35 (bs, 1H), 7.32-7.28 (m, 1H), 7. 12-7.06 (m, 2H), 6.35 (s, 1H), 2.82-2.78 (m, 2H), 2.39 (td, *J* = 5.4 and 7.1 Hz, 1H), 2.14 (td, *J* = 5.2 and 7.5 Hz,

1H). ^{13}C NMR (101 MHz, DMSO) δ 171.19, 134.86, 131.79, 127.96, 126.41, 119.58, 87.77, 85.74, 69.81, 44.48, 36.26, 29.84. C-3 aryl carbon not observed. High resolution mass spectrometry (HRMS) (Electrospray Ioniziation (ESI)+): m/z calculated for $C_{11}H_{13}O_3N_4$ B(boronic acid)+ 260.1075, found 260.1081.

Step 2: (R)-6-(3-((3-hydroxy-1-methyl-2-oxopyrrolidin-3-yl)ethynyl)phenyl)-4-methoxypicolinamide ***(NIK SMI1)***.

A 500 mL flask under inert atmosphere of nitrogen was charged with ethanol (300 mL), water (50 mL), 6-bromo-4-methoxypyridine-2-carboxamide (14.4 g, 62.325 mmol), potassium (*S*)-trifluoro(3-((3-hydroxy-1-methyl-2-oxopyrrolidin-3yl)ethynyl)phenyl)borate (20 g, 63 mmol, 1 equivalents), $Pd(PPh_3)_2Cl_2$ (4.37 g, 6.3 mmol, 0.10 equivalents), sodium carbonate (13.2 g, 125 mmol, 2.0 equivalents). The resulting solution was stirred overnight at 90°C. The solids were removed by filtration and discarded. The resulting solution was concentrated under vacuum. The residue was purified by flash column chromatography, (ethylacetate/petroleum ether 100/1). The crude product was purified by reverse-phase column chromatography with the following conditions: column, XBridge Prep C18 OBD Column, 50*200mm 25um~35nm; mobile phase, water with 10mmol NH_4HCO_3 and MeCN (20.0% MeCN up to 40.0% in 30 min); detector, UV 254/220nm. This purification resulted in 12.965 g (57%) of 6-(3-[2-[(3*R*)-3-hydroxy-1-methyl-2-oxopyrrolidin-3-yl]ethynyl]phenyl)-4-methoxypyridine-2-carboxamide as an off-white solid. LC-MS (ES, *m/z*): 366 $[M+H]^+$. ^1H NMR (400 MHz, DMSO-d_6) δ 8.36 – 8.30 (m, 2H), 8.30 – 8.28 (m, 1H), 7.71 (d, *J* = 2.5 Hz, 2H), 7.56 – 7.48 (m, 3H), 6.46 (s, 1H), 3.98 (s, 3H), 3.37 (dd, *J* = 7.2, 5.8 Hz, 2H), 2.81 (s, 3H), 2.48 – 2.43 (m, 1H), 2.20 (dt, *J* = 12.8, 7.2 Hz, 1H). ^{13}C NMR (101 MHz, DMSO) δ 171.28, 168.04, 166.31, 156.34, 152.68, 138.64, 132.97, 130.22, 129.53, 128.21, 122.78, 109.09, 107.48, 90.59, 84.35, 70.40, 56.37, 45.01, 36.54, 30.35. (Supplementary Fig. 15).
HRMS (ESI+): m/z calculated for $C_{20}H_{20}O_4N_3$ (M+H)+ 366.1448, found 366.1444

Synthesis of NIK SMI2

Step 1: 1-(4-iodo-2-pyridyl)indazole-3-carboxylic acid.

To a solution of methyl 1H-indazole-3-carboxylate (1.0 equivalents, 100 mg, 0.57 mmol) and cesium carbonate (2.0 equivalents, 370 mg, 1.14 mmol) in DMF (1.1 mL) was added 2-fluoro-4-iodo-pyridine (1.1 equivalents, 139 mg, 0.62 mmol). The reaction was heated to 100 ºC for 3 hours whereupon the reaction mixture was then acidified to pH 1 with 10% HCl and extracted with dichloromethane (3x). The organic layers were combined, dried over sodium sulfate and concentrated to afford 205 mg of crude 1-(4-iodo-2-pyridyl)indazole-3-carboxylic acid which was used in the next step without purification.

Step 2: (*R*)-1-(3-((3-hydroxy-1-methyl-2-oxopyrrolidin-3-yl)ethynyl)phenyl)-1*H*-indazole-3-carboxamide ***(NIK SMI2)***

Step 2a: Methyl 1-(3-bromophenyl)-1H-indazole-3-carboxylate.

In a 20-mL sealed tube purged and maintained with an atmosphere of oxygen, a suspension of methyl 1H-indazole-3-carboxylate (500 mg, 2.8 mmol), (3-bromophenyl)boronic acid (852 mg, 4.2 mmol, 1.5 equivalents), and copper(II) acetate (155 mg, 0.85 mmol, 0.3 equivalents) in DMF (5.7 mL)/pyridine (0.7 mL) was heated at 2 h at 90°C. The resulting mixture was concentrated under vacuum and diluted with 10 mL of water. The mixture was extracted with ethyl acetate (90 mL) and the organic layers were combined and dried over anhydrous sodium sulfate. The residue was purified by flash column chromatography (ethyl acetate/petroleum ether 1:20), to give 400 mg (43%) of methyl 1-(3-bromophenyl)-1H-indazole-3-carboxylate as a white solid. LC-MS (ES, m/z): 331, 333 $[M+H]^{+}$.

Step 2b: Methyl (R)-1-(3-((3-hydroxy-1-methyl-2-oxopyrrolidin-3-yl)ethynyl)phenyl)-1H-indazole-3-carboxylate.

In a 5-mL sealed tube purged and maintained with an atmosphere of nitrogen, a suspension of methyl 1-(3-bromophenyl)-1*H*-indazole-3-carboxylate (150 mg, 0.45 mmol), (3*R*)-3-ethynyl-3-hydroxy-1-methylpyrrolidin-2-one (126 mg, 0.91 mmol, 2.0 equivalents), $Pd(PPh_3)_2Cl_2$ (96 mg, 0.14 mmol, 0.30 equivalents) in triethylamine (1 mL) and dimethyl sulfoxide (1 mL) was irradiated with microwave radiation for 1.5 h at 90 ^{o}C. The reaction was quenched by addition of 10 mL of saturated brine and extracted with ethyl acetate (90 mL). The organic layers were combined, washed with 20 mL of saturated brine, and dried over anhydrous sodium sulfate.

Concentration under vacuum left a residue that was purified by a flash column chromatography, (ethyl acetate/petroleum ether 1:1), to give 130 mg (74%) of methyl 1-(3-[2-[(3*R*)-3-hydroxy-1-methyl-2-oxopyrrolidin-3-yl]ethynyl]phenyl)-1H-indazole-3-carboxylate as a yellow solid. LC-MS (ES, *m/z*): 390 $[M+H]^+$.

Step 2c: (R)-1-(3-((3-hydroxy-1-methyl-2-oxopyrrolidin-3-yl)ethynyl)phenyl)-1H-indazole-3-carboxamide

A solution of methyl 1-(3-[2-[(3R)-3-hydroxy-1-methyl-2-oxopyrrolidin-3-yl]ethynyl]phenyl)-1H-indazole-3-carboxylate (130 mg, 0.33 mmol) in methanol (saturated with ammonia, 20 mL) was stirred overnight at 40 ^{o}C. The resulting mixture was concentrated under vacuum. The crude product was purified by preparative high-pressure liquid chromatography (Prep-HPLC) with the following conditions: column, Xselect C18 19x150 mm; mobile phase, water with 0.05% NH_3 and MeCN (20.0% MeCN up to 35.0% in 8 min); detector, 254/220 nm. This resulted in 49.6 mg (40%) of 1-(3-[2-[(3*R*)-3-hydroxy-1-methyl-2-oxopyrrolidin-3-yl]ethynyl]phenyl)-1H-indazole-3-carboxamide as a white solid. LC-MS (ES, *m/z*): 375 $[M+H]^+$. ^{1}H NMR (400 MHz, DMSO-d_6) δ 8.31 (d, J = 8.2 Hz, 1H), 8.03 (s, 1H), 7.95 – 7.91 (m, 1H), 7.91 – 7.84 (m, 2H), 7.68 – 7.63 (m, 1H), 7.61 – 7.48 (m, 3H), 7.40 (dd, J = 8.1, 6.9 Hz, 1H), 6.52 (s, 1H), 3.37 (dd, J = 7.3, 5.5 Hz, 2H), 2.81 (s, 3H), 2.49 – 2.42 (m, 1H), 2.20 (dt, J = 12.7, 7.1 Hz, 1H). ^{13}C NMR (101 MHz, DMSO) 171.15, 163.99, 140.30, 140.00, 139.76, 130.76, 130.69, 128.54, 125.99, 123.98, 123.86, 123.78, 123.42, 123.01, 111.46, 91.48, 83.46, 70.39, 45.00, 36.44, 30.36 (Supplementary Fig. 16).

HRMS (ESI+): m/z calculated for $C_{21}H_{19}O_3N_4$ (M+H)+ 375.1452, found 375.1446
